# Direct in‐scope suction (DISS) ureteroscopy: techniques, outcomes and future directions

**DOI:** 10.1111/bju.16871

**Published:** 2025-08-05

**Authors:** Parth U. Patel, Michael Uy, Casey Dauw, Wilson Sui, Jeffrey Plott, William W. Roberts, Khurshid R. Ghani

**Affiliations:** ^1^ Department of Urology, Michigan Medicine University of Michigan Ann Arbor MI USA; ^2^ Department of Cardiac Surgery, Michigan Medicine University of Michigan Ann Arbor MI USA

**Keywords:** ureteroscopy, suction ureteroscopy, direct in‐scope suction, stone‐free rate, retrograde intrarenal surgery, laser lithotripsy

## Abstract

**Objective:**

To provide an overview of direct in‐scope suction (DISS) ureteroscopy, an emerging technology that integrates suction directly into the ureteroscope with the aim of enhancing stone clearance, improving visualisation, and reducing intrarenal pressure during ureteroscopic stone surgery.

**Methods:**

We performed a narrative review of the current literature and incorporated insights from the authors’ clinical experience using DISS ureteroscopy systems.

**Results:**

Direct in‐scope suction ureteroscopes are either single‐channel or dual‐channel. With single‐channel ureteroscopes, irrigation must alternate with suction. A dual channel allows synchronous irrigation and suction. The suction can be applied continuously or intermittently (alternating with passive drainage). By actively managing intrarenal pressure, DISS may lower the risk of infectious complications and inadvertent laser injury. Unlike traditional ureteroscopy, some DISS systems may reduce the need for a ureteric access sheath (UAS). Early clinical data demonstrate that DISS offers stone‐free rates comparable to standard ureteroscopy, with no increase in major complications. However, performance varies across devices, with trade‐offs related to scope size, flexibility, suction power, and risk of clogging or collecting system collapse. Larger‐calibre DISS ureteroscopes may face deflection limitations in tight calyces as well as the need for a UAS. Refinements in single‐use platforms are addressing these challenges. DISS may be especially beneficial in patients with moderate‐to‐large stone burdens by enabling more efficient fragment evacuation and reducing the need for secondary procedures. Emerging technologies – such as integrated suction‐laser tools and pressure‐monitoring systems – promise to expand DISS capabilities further.

**Conclusion:**

Direct in‐scope suction ureteroscopy represents a significant evolution in endourological practice by addressing key limitations of standard ureteroscopy, namely, fragment management, intrarenal pressure control, and visualisation. There remains a need for high‐quality level 1 evidence. With ongoing innovation, future DISS systems may offer complete stone clearance.

**Abbreviations**
DISS: direct in‐scope suction
FANS: flexible and navigable suction
PCNL: percutaneous nephrolithotomy
RF: residual fragment
SFR: stone‐free rate
UAS: ureteric access sheath

## Introduction

Ureteroscopy is the primary intervention for many upper urinary tract stones, but conventional ureteroscopy has limitations in achieving complete stone clearance [1]. Stone fragment extraction with baskets can be tedious, prolonging operating time with no significant improvement in stone‐free rates (SFRs) [2]. The laser ‘dusting’ technique, especially with high‐power holmium or thulium fibre lasers, fragments stones into fine dust to obviate basket retrieval [3]. While dusting can improve efficiency, it often causes a ‘snow globe’ effect of suspended particles that impair visibility [4]. Thus, despite advanced lasers, residual fragments (RFs) and poor intra‐operative visibility remain challenges in ureteroscopy.

Direct in‐scope suction (DISS) ureteroscopy is an emerging technology, developed to address these challenges by integrating active suction directly into the ureteroscope [5]. By aspirating stone dust and small fragments during laser lithotripsy, the aim of DISS is to improve visualisation, enhance fragment clearance, reduce dependency on baskets and ureteric access sheaths (UASs), and better regulate intrarenal pressure [6].

There are two types of DISS system: single‐channel and dual‐channel. With single‐channel devices, irrigation must alternate with suction. Dual‐channel devices can provide synchronous irrigation and suction, where suction can be applied continuously or intermittently (alternating with passive drainage). These different approaches require an external vacuum source connected via tubing to the ureteroscope.

Early clinical experience suggests that DISS can mitigate the ‘snow globe’ effect and potentially improve the SFR and operative efficiency by actively removing debris during ureteroscopy [7]. Given the rapid development of this technology, we conducted this review to provide an updated overview of DISS ureteroscopy techniques, commercially available devices, clinical outcomes, safety profile, and future innovations.

### Suction Adaptations in Conventional Ureteroscopy

**Fig. 1:**
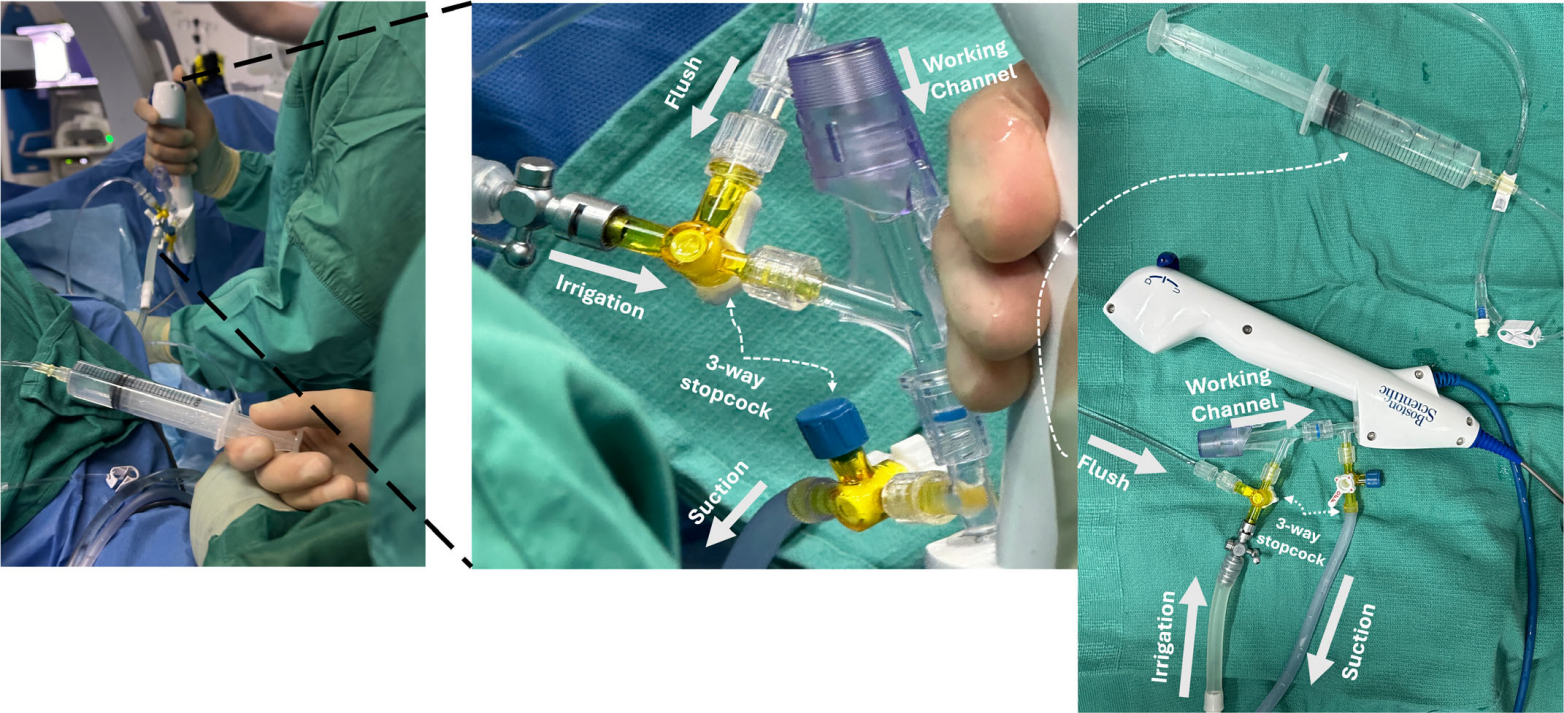
The use of three‐way stopcocks allows surgeons to irrigate, flush/aspirate and suction debris from the collection system during active ureteroscopy. The surgeon can close the irrigation fluid and open the channel to flush debris using the first three‐way stopcock. Additionally, with the irrigation fluid shut off, the second three‐way stopcock can be turned to allow the suction of debris via the working channel of the ureteroscope. This is a key advance in suction adaptations during conventional ureteroscopy.

Initial attempts to incorporate suction into ureteroscopy involved adaptations of existing ureteroscopes. In the early 2000s, a pilot study with a rigid ureteroscope used an automated irrigation/suction system to control pressure and flow, demonstrating improved stone removal compared to standard irrigation [8]. Translating continuous inflow–outflow systems to flexible ureteroscopes was challenging. A key advance was three‐way stopcock adaptors on the working channel port, enabling alternating suction and irrigation without modifying the ureteroscope (Fig. 1). Surgeons used a hand syringe or suction tubing to aspirate fluid and debris periodically during laser lithotripsy, often pausing irrigation to apply suction. Schneider et al. [9] showed *in vitro* that intermittent aspiration through a standard 3.6‐F channel evacuated stone fragments more effectively than irrigation alone. While these do‐it‐yourself modifications improved visibility, they were limited by the small calibre of conventional scope channels. Fragments could easily clog the channel, necessitating frequent scope removal to clear obstructions. Moreover, repeatedly switching between irrigation and suction interrupted the procedure and could prolong operating time [10].

#### Single‐Lumen Flexible Ureteroscopes with Suction Attachments

To enhance intermittent suction capability with a single‐channel ureteroscope, accessory devices have been developed. One example is the new GLITZ system, a lightweight 100‐g suction attachment that is mounted on a standard flexible ureteroscope handle [7]. The GLITZ device connects to an external irrigation–aspiration unit and features a finger trigger; when depressed, this diverts fluid flow from irrigation to suction, aspirating through the ureteroscope's working channel. Releasing the trigger resumes irrigation flow. This design provides on‐demand suction while automatically pausing irrigation to prevent excessive intrarenal pressure – a built‐in safety mechanism that halts flow if a blockage occurs. The GLITZ essentially converts any single‐channel digital ureteroscope (typically a ~7.5–9‐F ureteroscope with a 3.6‐F working channel) into an alternating irrigation and suction device.

An early clinical evaluation of the GLITZ system via a 29‐patient multicentre audit reported that surgeons found the device easy to use in 93% of cases, achieving complete dust aspiration in 62% [7]. At 30‐day follow‐up, 75.9% of patients had no RFs on CT. Issues were noted, such as the attachment becoming dislodged in ~24% of cases (likely during scope manipulation). Complications were self‐limited haematuria and a 10.3% rate of transient postoperative fever. Overall, an add‐on suction device, such as the GLITZ device, demonstrates that even within the constraints of a single‐lumen ureteroscope, active aspiration and fragment clearance is possible in flexible ureteroscopy.

#### Dual‐Lumen Ureteroscopes for Synchronous Suction

Another strategy is to build flexible ureteroscopes with two working channels – one for irrigation and one for outflow. The Wolf Cobra (reusable fibre‐optic scope), Cobra Vision (reusable digital) and single‐use hybrid semirigid/flexible Richard Wolf Disposable Ureteroscope (RIWO D‐URS), exemplify this dual‐lumen design. The Wolf Cobra has an 11.1‐F outer diameter with twin 3.3‐F channels, whereas the Cobra Vision is slimmer, with a 9.9‐F outer diameter and one 2.4‐F and one 3.6‐F working channel [11]. The D‐URS has a 9.0‐F outer diameter and contains a 3.6‐F working channel and separate 1.65‐F laser channel, and allows fluid to drain through the interstitial space of the ureteroscope shaft. In practice, for each of these configurations, one channel can be dedicated to irrigation and the other to drainage, enabling continuous circulation that produces improved intrarenal pressure and flow rate compared to single‐channel ureteroscopes and may prevent the abrupt pressure spikes or collapses that can occur with intermittent suction [12, 13].

Ureteroscopes with two true lumens enable synchronous suction during laser lithotripsy without interrupting irrigation. In a porcine model, Jiang et al. [11] showed 94% stone clearance with a dual‐lumen ureteroscope vs 65% with a single‐channel ureteroscope, with no increase in operating time. The main drawback is size: the 11.1‐F Cobra may require ureteric dilatation or a UAS, while the slimmer 9.9‐F Cobra Vision has a smaller 2.4‐F suction channel that may limit fragment clearance. Nevertheless, these ureteroscopes pioneered integrated suction, laying the groundwork for modern DISS systems.

### Dedicated DISS Ureteroscopes (Commercial Devices)

**Fig. 2:**
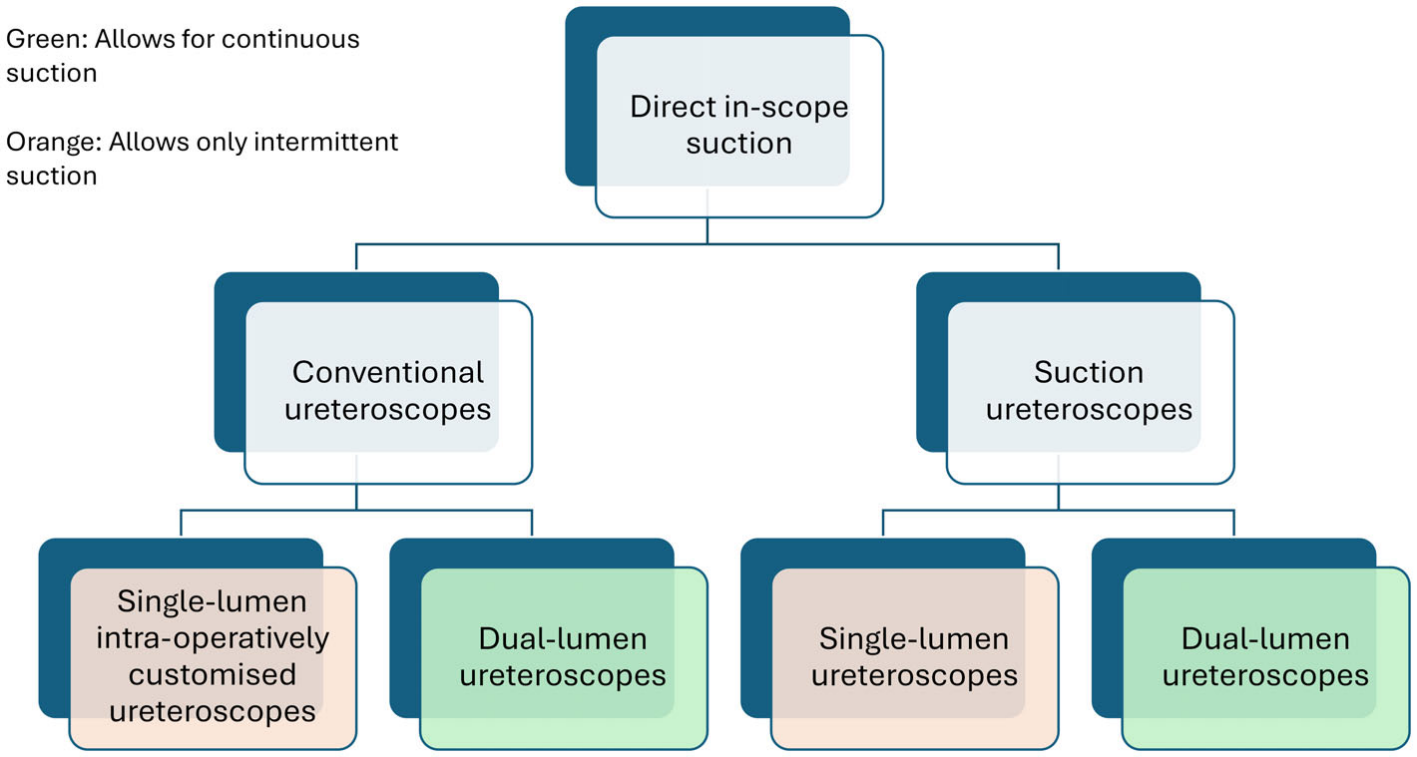
Different designs of commercially available suction ureteroscopes.

Fully integrated DISS ureteroscopes represent a significant innovation – these single‐use digital scopes are purpose‐built with suction capability. They incorporate design features to aspirate fragments without external adapters, aiming to streamline the procedure [7, 14, 15, 16, 17, 18]. Two main categories have emerged: intermittent suction scopes that share a single lumen for both irrigation and suction (requiring alternating flow), and synchronous suction and irrigation scopes that have separate channels for irrigation and drainage. Figure 2 illustrates the different designs of the commercially available suction ureteroscopes.

#### Single‐Lumen Digital Scopes with Intermittent Suction

The Pusen DISS flexible ureteroscopes (Pusen Medical, China) are among the first commercially available single‐use ureteroscopes with in‐scope suction. The initial model (PU3033A‐HD) is a 7.5‐F digital ureteroscope with a 3.6‐F working channel [18]. It features a suction port on the handle that connects to suction tubing, effectively allowing the working channel to be used for either irrigation or aspiration. During lithotripsy, the surgeon depresses a button and must also stop irrigation to effectively apply suction, thereby intermittently aspirating stone dust and small fragments through the channel.

Early clinical evaluations of this ureteroscope have shown encouraging results. In a multicentre feasibility study (57 cases), Nedbal et al. [16] reported an initial SFR of 84.2% (no fragments >2 mm on postoperative imaging). Surgeons rated the scope highly in terms of manoeuvrability, visibility, and overall satisfaction, with 94.7% agreeing that the integrated suction improved the procedure. Another series by Geavlete et al. [15] compared conventional flexible ureteroscopy to the Pusen DISS scope: using DISS with a UAS improved the SFR from 82.9% to 97.2% in their cohort (no RF >3 mm at 3 weeks). These findings suggest that intermittent in‐scope suction could boost stone clearance.

However, first‐generation single‐lumen DISS ureteroscopes have limitations. Because irrigation and suction share one channel, the surgeon must frequently alternate between the two functions, and the 3.6‐F channel diameter restricts the fragment size that can be aspirated (approximately <250‐μm particles, based on *in vitro* testing) [9]. Notably, pressing the suction button alone will only draw irrigation fluid. To enable suction from the tip of the ureteroscope, the button must be pressed and the irrigation stopcock must be turned off.

Recognising these limitations, a larger model has been developed. The Pusen PU400A has a 9.2‐F outer diameter and a wider 5.1‐F working channel [14, 19]. The larger channel is designed to evacuate bigger fragments and increase flow rates. A recent *in vitro* analysis found that, while a 3.6‐F channel can reliably aspirate stone dust up to approximately 0.25 mm in size, the 5.1‐F channel should be able to aspirate larger fragments [14].

Paradoxically, early experiments suggest bigger is not always better. Tsaturyan et al. [19] tested 7.5‐F vs 9.2‐F Pusen DISS ureteroscopes in an *ex vivo* model and found the smaller ureteroscope had greater success in using suction to reposition 3–4‐mm stone pieces, especially from lower pole calyces. The 7.5‐F ureteroscope repositioned 3–4‐mm fragments in 40%–80% of trials, while the 9.2‐F scope performed worse (often 0%–20% in the lower pole). Its stronger suction caused faster system collapse (~4 vs ~6–7 s), limiting fragment retrieval. This underscores the trade‐off between suction strength, scope size, and flexibility – larger channels improve flow but reduce deflection and hasten collapse in tight calyces. Newer DISS ureteroscope designs aim to enhance clearance while minimising clogging and collapse. Despite these challenges, Pusen DISS has shown acceptable operating times and safety in clinical use.

#### Ureteroscopes with Integrated Synchronous Suction

Synchronous suction and irrigation DISS systems incorporate a dedicated aspiration lumen separate from the irrigation channel. The flagship device in this category is the Calyxo CVAC® System (Calyxo, Inc.), a next‐generation ureteroscope inspired by the earlier SURE™ (steerable ureteroscopic renal evacuation) vacuum‐assisted lithotripsy concept [20, 21]. The Calyxo DISS ureteroscope is a disposable, second‐generation digital scope (~11.9 F) with dual channels, including a 6.9‐F suction/working channel. Used with a vacuum pump and collection canister, it aspirates stone fragments during ureteroscopy. Four angled microjet nozzles near the tip stir up debris for easier suction. Active suction is activated by fully depressing the handpiece trigger (passive suction occurs when not fully depressed). The system can evacuate fragments up to ~2 mm in size.

Early clinical evaluation of the predecessor of the Calyxo synchronous suction system (first‐generation CVAC/SURE), via a steerable catheter method has shown promising outcomes, particularly for large stone burdens. The ASPIRE trial, a prospective randomised study, compared vacuum‐assisted ureteroscopy using the first‐generation CVAC/SURE steerable catheter vs conventional ureteroscopy with basket extraction for renal stones [20]. In this technique, after ureteroscopy for renal calculi, a steerable catheter (7.5 Fr) is advanced through a UAS. Using retrograde pyelography under fluoroscopic guidance, the catheter tip is navigated into individual calyces to irrigate and aspirate residual stone fragments. This method is performed without direct endoscopic visualisation and relies on fluoroscopy for catheter positioning. At 30 days post‐procedure, the two groups had similar SFRs by CT (no fragments >2–4 mm), confirming the non‐inferiority of this method. However, the steerable catheter achieved greater stone clearance: mean volume reduction was 96.9% vs 92.9% (*P* = 0.036), and residual volume was fivefold lower (14 mm^3^ vs 70 mm^3^; *P* = 0.012). The efficacy of the DISS CVAC steerable catheter remained consistent with larger stones, unlike standard ureteroscopy. Only 4% of patients with the steerable catheter needed secondary procedures vs 9% in controls, despite an average stone size >1.5 cm. A subsequent multicentre study by Stern et al. [21] reported 95.8% of large (≥10 mm) stones cleared ≥80% in cases using the SURE/CVAC aspiration catheter, with one third showing no RFs on follow‐up CT. No device‐related complications were observed in these studies. These results suggest that synchronous suction and irrigation, as has been incorporated in the second‐generation CVAC DISS ureteroscope, may approach acceptable stone‐free outcomes for intermediate‐sized stones.

The Calyxo DISS ureteroscope has key limitations. Its large size (11.9 F) requires a 12/14‐F UAS, and while the wide suction channel enhances fragment removal, it can clog, necessitating flushing or scope removal and prolonging the case. More concerning is unrecognised suction blockage: if irrigation continues despite a clog, intrarenal pressure may rise, negating safety benefits. The large ureteroscope may also limit passive drainage around the sheath. Unlike systems with separate suction sheaths that vent excess pressure, the Calyxo system lacks built‐in pressure relief, increasing the risk of complications such as urosepsis or, rarely, fornix rupture [22, 23, 24]. In a recent case in the United States, a death was reported in the US Food and Drug Administration Manufacturer and User Facility Data Experience system [25]. Although rare, these events highlight the need for use of a careful technique with DISS. The larger ureteroscope may also limit deflection in tight calyces. However, as technology advances, synchronous suction ureteroscopes, such as the CVAC system, offer the potential for improved stone clearance in a single session, reducing the need for multiple ureteroscopy procedures, or percutaneous nephrolithotomy (PCNL), which confers risks of access‐related complications.

Table 1 summarises the key features of representative DISS ureteroscope systems. Different design approaches (single vs dual lumen) entail trade‐offs in size, suction power, and fragment clearance capability.

**Table 1:** Comparison of direct in‐scope suction ureteroscopes and suction devices.

Device/system	Design and use	Scope size	Working channel	Suction mode	Notable performance
Conventional URS + adapter	Standard reusable flexible scope + two‐way stopcock for syringe or wall suction. Used intermittently	8.5–9.5 F (typical flexible URS) [7]	~3.6 F, single channel	Intermittent (manually alternating irrigation/suction)	Improves visualisation; fragment clogging common
GLITZ suction accessory [6, 7]	Trigger attachment to standard URS handle, connects to suction unit. Intermittent on‐demand suction	Adopts host ureteroscope size (e.g. 7.5–9‐F single‐use URS)	~3.6 F (host ureteroscope channel)	Intermittent (trigger‐controlled alternating irrigation/suction)	96.6% SFR (2‐mm fragments) at 30 days; 75.9% completely stone‐free. 10% fever, no sepsis [6, 7]
Wolf Dual Lumen Cobra [11]	Reusable dual‐channel digital URS. Continuous suction with separate irrigation	11.1‐F outer diameter	Two channels (each 3.3 F)	Continuous flow	94% vs 65% clearance in *ex vivo* test vs single‐lumen URS. Large size may require dilatation [11]
Wolf Dual Lumen Cobra Vision [11]	Reusable dual‐channel digital URS (newer slim version). Continuous suction	9.9‐F outer diameter	Two channels (2.4 F + 3.6 F)	Continuous flow	Improved fluidics with smaller calibre. Channels may limit fragment size (2–3 mm). Limited clinical data available [11]
Pusen DISS 7.5 F (PU3033) [17]	Single‐use digital flexible URS with suction port. Typically used with UAS	7.5‐F outer diameter	3.6‐F single channel	Intermittent (alternate irrigation/suction)	84% one‐session SFR (no >2‐mm fragment) Suction effective for dust <250 μm. Highly rated manoeuvrability [14, 15]
Pusen DISS 9.2 (PU400A) [13]	Next‐generation, single‐use DISS ureteroscope (wider channel). For larger fragments	9.2‐F outer diameter	5.1‐F single channel	Intermittent (alternate irrigation/suction)	Aims to aspirate particles >250 μm up to ~4 mm. Strong suction but faster calyx collapse noted *ex vivo*. Clinical data pending [18]
Calyxo CVAC System [19, 20]	Single‐use (or limited‐reuse) digital DISS URS with vacuum and irrigation integration. Uses external vacuum pump	~11.9‐F outer (with sheath ~13 F)	6.9‐F suction channel (+ separate irrigant channel)	Synchronous suction and irrigation (separate channels)	Evacuates fragments up to 2 mm. Larger URS

Specifications and performance metrics for major DISS technologies.

DISS, direct in‐scope suction; SFR, stone‐free rate; UAS, ureteric access sheath; URS, ureteroscope.

## Special Applications of DISS in Ureteroscopy

Beyond routine cases, DISS ureteroscopy offers advantages in challenging scenarios. Lower‐pole renal stones have long been problematic for conventional ureteroscopy because gravity hinders fragment clearance from dependent calyces [26, 27]. Even with patient repositioning (e.g. prone or reverse Trendelenburg), small fragments tend to accumulate in lower‐pole calyces during surgery. Suction may increase the SFR for lower‐pole stones by removing fragments that would otherwise remain trapped [11]. A recent retrospective study compared a flexible and navigable suction (FANS) UAS vs sheathless DISS for 2–3‐cm lower‐pole stones: both methods achieved high overall clearance (83%–88% after staged procedures), confirming that DISS is effective even for these challenging stones [6]. The FANS approach cleared fragments slightly faster (shorter operating time) in that series, but required placement of a UAS, whereas the DISS approach avoided a UAS at the cost of a modestly longer surgery. In either case, applying suction significantly aids in managing lower‐pole calculi, reducing the need for secondary procedures.

Direct in‐scope suction is also beneficial for large stone burdens that would otherwise require multiple ureteroscopy sessions or alternative treatments. By clearing the visual field of stone dust and small fragments, DISS allows the surgeon to increase the amount of time with laser fragmentation, without too many pauses [16]. This improves efficiency in debulking big stones and ensures that once a stone is pulverised, the remnants are promptly evacuated rather than accumulating. Studies have reported that using suction during ureteroscopy for large (2‐cm) renal stones can achieve stone‐free outcomes comparable to those of mini‐PCNL in some cases [28]. The suction not only evacuates fragments but also flushes out blood and stone debris, maintaining a clear endoscopic view, which is crucial when tackling sizeable calculi. DISS is emerging as a viable option for select patients with large stones, especially those who are poor candidates for PCNL (e.g. those on anticoagulation or with complex anatomy).

Another potential application is the management of steinstrasse. A recent case series described using a specialised vacuum‐assisted ureteroscope system to treat steinstrasse in 22 patients, achieving 100% immediate clearance of the ureteric stone column with only minor complications [29]. This underscores that suction technology can be adapted to remove extensive stone debris, even in the ureter. As these examples illustrate, DISS can expand the capabilities of ureteroscopy into territories that were previously challenging – lower‐pole stones, large stone burdens, and complex multi‐fragment scenarios – by improving fragment management and visualisation in real time.

## Clinical Outcomes with DISS Ureteroscopy

The primary goals of integrating suction into ureteroscopy are to improve SFRs, maintain acceptable operating times, and enhance patient safety. Below we summarise the clinical outcome data from key studies of DISS ureteroscopy, highlighting stone clearance, operative efficiency, and complications.

### Stone‐Free Rates and Fragment Clearance

Across the early studies, DISS ureteroscopy has demonstrated high SFRs, often superior to those achieved with conventional ureteroscopy in comparative analyses. Because definitions of ‘stone‐free’ vary, it is important to note what cut‐offs were used for RFs.

#### Dust Clearance Efficacy

*In vitro* experiments provide proof of concept that DISS outperforms passive irrigation in removing fine particles. Madden et al. [19] showed that a suction‐integrated single‐use ureteroscope achieved 100% clearance of dust <250 μm significantly faster than manual irrigation methods. This shows the ability of DISS to evacuate ‘dust’ that would otherwise linger and obscure vision.

#### Initial Clinical Series

The first clinical evaluations of DISS reported excellent SFRs. Nedbal et al. [16] found an 84.2% SFR (no RF >2 mm on X‐ray/ultrasonography/CT) after a single procedure using the 7.5‐F Pusen DISS scope. Geavlete et al. [15] directly compared standard ureteroscopy vs DISS ureteroscopy (with and without a suction UAS) in a multicentre trial. The highest success was achieved with DISS + suction UAS, yielding a 97.2% SFR (no RF >3 mm at 3 weeks on X‐ray or ultrasonography) vs 82.9% with conventional ureteroscopy using a suction UAS (*P* < 0.01). Notably, even DISS with a conventional UAS (no active suction) improved the SFR to ~90%, suggesting that in‐scope suction alone confers benefit.

Overall, the early evidence suggests that the active suction in DISS may translate to higher SFRs. By removing debris, DISS reduces the likelihood of RFs being left behind. Table 2 summarises outcomes from selected studies. In summary, the active fragment evacuation enabled by DISS translates into improved stone‐free outcomes in both experimental and clinical settings.

**Table 2:** Clinical outcomes in selected direct in‐scope suction ureteroscopy studies.

Study (year)	DISS method	SFR	Operating time	Complications
Nedbal et al. (2024) [16]	Pusen 7.5‐F DISS ureteroscope (single‐use); multicentre feasibility	84.2% SFR (no RF >2 mm on imaging) after single URS	~50 min (median) – comparable to standard URS (qualitative)	Minor only; no major complications reported. Surgeons rated suction helpful in 94.7%
Geavlete et al. (2024) [15]	Pusen DISS vs conventional URS (three arms); multicentre comparative	97.2% SFR with DISS + suction sheath (highest) vs 82.9% with standard URS (no RF >3 mm)	53 min (DISS) vs 50 min (standard URS), difference not significant	Similar peri‐operative outcomes between groups; no significant safety differences noted
Gauhar et al. (2025) [7]	GLITZ suction accessory on 7.5‐F ureteroscope; multicentre audit	75.9% completely stone‐free; 96.6% including 2‐mm fragments at 30 days	62.3 min mean (GLITZ) vs ~60 min historical URS. Not significantly different	10.3% fever; no ureteric injuries. Device dislodgement in 24%
Yıldırım et al. (2025) [30]	Sheathless DISS (Pusen 7.5) vs FANS sheath; 2–3‐cm lower‐pole stones	After one URS: 46.4% SFR DISS vs 62.5% FANS (*P* = 0.16); after two URS: 82.1% vs 87.5% (*P* = 0.41; SFR defined as no RF >4 mm). Complete SFR (zero RF) after 2 sessions ~75% DISS vs 84% FANS (n.s.)	79.1 ± 11.7 min (DISS) vs 71.8 ± 11.5 min (FANS), *P* = 0.026. Longer time for DISS attributed to dusting mode needed for large stone	Post‐op fever: 10.7% DISS vs 9.3% FANS (n.s.). No ureteral strictures or serious complications in either group. DISS avoided UAS usage
Matlaga et al. (2024 ASPIRE) [20]	SURE procedure with Calyxo CVAC vs standard URS; RCT for 7–20 mm stones	48% vs 49% complete SFR (no RF >0 mm) at 30 days (non‐inferior). *Effective* SFR (no RF >2 mm) high in both (~92–95%, n.s.). Stone volume clearance: 97% DISS vs 93% URS (*P* = 0.036)	82 min (mean) DISS vs 79 min URS (n.s., not reported as significantly different). Learning curve ongoing	No differences in complications; low overall morbidity. DISS group required fewer secondary procedures

DISS, direct in‐scope suction; FANS, flexible and navigable suction (ureteric access sheath); n.s., not significant; RCT, randomised controlled trial; RF, residual fragment; SFR, stone‐free rate; URS, ureteroscope.

### Operating Time

A concern associated with adding suction to ureteroscopy is the potential for longer operating times. However, by clearing fragments in real time, DISS may reduce the need for basket retrieval and flushing, potentially shortening procedures. While managing suction settings could introduce delays, the limited evidence shows that operating times with DISS are generally comparable to those with standard ureteroscopy, with any differences likely attributable to stone complexity rather than the suction itself.

Several studies have reported equivalent operating times for DISS and traditional ureteroscopy. Geavlete et al. [15] saw no significant difference (mean ~ 50–53 min in both arms) when comparing Pusen DISS to regular ureteroscopy for ~1‐cm stones. Jiang et al. [11] also found no statistical difference in mean operating time between dual‐lumen suction ureteroscopes and single‐lumen ureteroscopes in an *ex vivo* setting. In the multicentre Pusen DISS study, surgeons subjectively felt that the enhanced visibility helped streamline the procedure and gave the device a high score (4.15/5) for potentially reducing operating time, reporting a mean operating time of 50 min [16].

Conversely, some reports noted longer case times with DISS, but typically in contexts of treating larger stones. Gauhar et al. [6] observed a mean operating time of 80 min for DISS cases vs 47.5 min for non‐suction ureteroscopy, but the DISS group's stones were nearly twice the size on average (22 vs 13 mm) and achieved an equivalent final SFR. In that sense, DISS allowed surgeons to tackle more complex stones in one session, albeit with a longer surgery – a worthwhile trade‐off. The recent FANS vs DISS comparison for large lower‐pole stones showed the DISS (sheathless) group had a mean operating time that was ~ 7 min longer than that of the suction sheath group (79 vs 72 min) [30]. The authors attributed the difference to DISS requiring meticulous dusting to fit fragments through the scope, while the FANS sheath allowed the removal of larger chunks, speeding fragmentation. This shows how laser strategy (dusting vs chunking) interacts with suction technology – DISS favours dusting, which can be time‐consuming for large stones but eliminates the need for basket retrieval. Despite longer cases with DISS, both groups achieved similar outcomes without added morbidity.

### Safety and Complications

Safety is critical with any new technology. DISS ureteroscopy brings in unique considerations around suction. While concerns exist about mucosal trauma or high vacuum pressures, clinical reports have not shown increased major complications. In fact, by controlling intrarenal pressure, DISS may help reduce certain risks.

#### Intra‐operative Visualisation and Trauma

Synchronous or alternating irrigation and suction improves visibility by clearing blood, potentially reducing laser injury. However, strong suction can cause transient mucosal bleeding or petechiae from calyceal or ureteric wall collapse. Gauhar et al. [7] reported ‘contact bleeding’ from suction pulling the scope tip against urothelium or sudden vessel decompression – events that were self‐limited and clinically insignificant. Overall, DISS has not increased ureteric injury rates and may even reduce trauma by eliminating the need for large access sheaths in some cases.

#### Intrarenal Pressure and Infectious Risk

One of the touted advantages of DISS is better control of intrarenal pressure. High intrarenal pressure during ureteroscopy can force bacteria into systemic circulation (pyelovenous backflow), contributing to post‐ureteroscopy fever or sepsis [24]. Synchronous aspiration in DISS is used to try to actively lower intrarenal pressure, and studies have demonstrated reduced pressure transmission with suction devices. Du et al. [31] showed that adding suction (via a specialised sheath) reduced the frequency of post‐ureteroscopy infectious complications in patients with large ureteric stones. In DISS trials to date, sepsis rates have been very low. In the study by Yıldırım et al. [30] both suction groups had similar mild fever rates (~10%) and no severe infections. By preventing prolonged high intrarenal pressure, DISS likely contributes to these favourable outcomes.

## Future Directions and Innovations

The integration of suction into flexible ureteroscopy is still in its relative infancy, and ongoing innovations promise to refine and expand DISS capabilities. Continuous suction flow scopes, where synchronous suction and irrigation are continually active, are not yet available for commercial use in Europe or North America. Ghani and Plott [32] reported the results of an experimental continuous flow 8.5‐F ureteroscope that has a 3.6‐F channel for suction and smaller outflow channels at the tip for continuous irrigation. Irrigation flows through the entire interstitial space (dead space) of the scope, and exits out through small tip openings. In *in vitro* testing with calcium stones, continuous suction through this prototype ureteroscope reduced the amount and size of RFs.

One foreseeable advancement is the automation and synchronisation of suction with laser lithotripsy. Future ureteroscopes may have laser systems linked to suction control such that activation of the laser automatically triggers aspiration. This would ensure that each burst of laser energy (which creates stone dust) is immediately accompanied by suction, essentially removing debris in real time without the surgeon having to manually toggle suction [5]. This kind of intelligent energy‐suction coupling could streamline the procedure and free the surgeon to focus solely on stone targeting, improving efficiency.

Another area of development is smart pressure‐sensing and regulated suction. Embedding micro pressure sensors at the ureteroscope tip or within the collection system could allow for dynamic feedback control of suction intensity [33, 34]. For instance, if intrapelvic pressure begins to drop too quickly (risking collapse), the system could automatically taper the suction to maintain a safe threshold. Conversely, if pressure rises (e.g. due to clogging or increased irrigation), the system could boost suction or alarm the user. Such closed‐loop systems, potentially augmented by machine learning algorithms, might optimise irrigation and aspiration rates on the fly to preserve a clear field while preventing any extremes of pressure that could injure the kidney. This would enhance safety, particularly for synchronous suction devices, by diminishing the risk of over‐ or under‐pressurisation.

Device miniaturisation and versatility are also anticipated. We may see suction‐enabled ureteroscopes in smaller calibres that retain effective flow. Achieving a high flow rate in a small lumen might involve novel lumen geometries or ultra‐slippery coatings to reduce resistance. In the future, surgeons might tailor the combination of tools – for example, using a suction UAS in conjunction with a suction ureteroscope for maximum evacuation (some have dubbed this ‘dual‐suction ureteroscopy’). Indeed, the highest SFR in Geavlete et al. [15] came from using both a DISS ureteroscope and an aspiration‐assisted UAS together.

Potential innovations include integrating laser fibres or baskets directly into suction channels, enabling lithotripsy within a controlled aspiration stream and simplifying workflow. Concepts such as coaxial lasers or ‘vacuum baskets’ remain experimental but may complement DISS with fragmentation‐resistant accessories that allow suction.

Looking ahead, more clinical data and long‐term outcomes will clarify which stone sizes benefit most from DISS, establish the cost‐effectiveness of single‐use suction scopes, and determine the learning curves associated with DISS adoption. If health economic studies suggest higher one‐session clearance with DISS, this could reduce overall costs by avoiding repeat procedures. The future could be a shift in guidelines toward favouring DISS for patients with moderate‐sized stones, who are currently recommended for PCNL.

In conclusion, DISS ureteroscopy marks a major advance in endourology by tackling fragment management via vacuum technology to improve stone clearance and outcomes. As devices become smarter, smaller, and more integrated, DISS is poised to become standard in ureteroscopic stone surgery. However, there is a need to standardise *in vitro* assessments and determine how outcomes are defined in the laboratory, with a consensus around endpoints that can lead to ongoing refinements. Furthermore, clinical performance should be evaluated through high‐quality randomised controlled trials. It is possible that future DISS systems will bring us closer to truly complete stone clearance in a single session – fulfilling the goal of leaving no stone behind.

## Disclosure of Interests

Parth Patel: none. Michael Uy: none. Casey Dauw: receives grant funding from Blue Cross Blue Shield of Michigan and PCORI. Has consulting activity with Boston Scientific, Karl Storz and Ethicon. Wilson Sui: has consulting activity with Boston Scientific and Karl Storz. Jeffrey Plott: receives grant funding from Coulter Translational Program. William Roberts: receives grant funding from Boston Scientific, and has consulting activity with Boston Scientific and Calyxo. KR Ghani: receives grant funding from Coloplast, Boston Scientific and Coulter Translational Program, and has consulting activity with Boston Scientific, Coloplast, Karl Storz, Ambu and Olympus.

## Funding

No funding was received for this work.
